# The role of men in antenatal care and preventing HIV transmission from mothers to infants in Gambella region, Ethiopia

**DOI:** 10.1371/journal.pgph.0000879

**Published:** 2022-08-29

**Authors:** Mirgissa Kaba, Michelle R. Kaufman, Andrea Ruff

**Affiliations:** 1 School of Public Health, Addis Ababa University, Addis Ababa, Ethiopia; 2 Bloomberg School of Public Health, Johns Hopkins University, Baltimore, Maryland, United States of America; Institute of Tropical Medicine: Instituut voor Tropische Geneeskunde, BELGIUM

## Abstract

Prevention of vertical transmission of HIV has evolved over the past two decades. Although public health agencies advocate for male involvement in such interventions, their role in the prevention of vertical transmission of HIV remains limited. This study aims to explore the role of men in antenatal care and prevention of vertical transmission of HIV in the Gambella region of Ethiopia. The study was conducted among the Nuer and Anuak communities in Gambella region. Participants included female antenatal care attendees at two health centers, men who were husbands, local health extension workers of the catchment villages, and health care providers and managers. Data were collected using focus group discussions, in-depth interviews, and key informant interviews. Recordings were transcribed, coded, and analysed using thematic approaches. MAXQDA version 11 was used to facilitate data organization and reduction. Findings showed that men in Gambella are not expected to accompany their wives to health facilities or encourage them to visit the facilities in connection to pregnancy. Participants reported that men rarely visit health facilities with their partners, and they are largely unaware of the connection between antenatal care during pregnancy and prevention of mother to child transmission of HIV. Participants indicated that women’s attendance at health facilities during pregnancy is not a common practice, and if they do visit them in connection to pregnancy there may be suspicion she has HIV. In this cultural context, men do not have a role in the health care of their wives during pregnancy. Men’s engagement may be further lessened by the common perception that visiting a health facility in connection to pregnancy is associated with HIV.

## Introduction

Despite nearly 40 years of extensive prevention, care, and treatment interventions, HIV is still a significant public health threat [1, 2]. Globally, 37.7 million people live with the virus, 73% of whom are on antiretroviral treatment (ART). Of these, over two-thirds are from sub-Saharan Africa [3]. Children ages 14 and under account for 5% of existing HIV infections and 9% of new ones; in 2019, 84% of new infections in children occurred in sub-Saharan Africa [4]. Reducing or stopping such infection rates among children requires investment in prevention of mother-to-child transmission (PMTCT), which has been well-documented to prevent vertical transmission and keep the mother and infant healthy [5, 6].

Given the need for PMTCT, the World Health Organization (WHO) has introduced a program that helps prevent vertical transmission of HIV during pregnancy, delivery, and breastfeeding [6]. In WHO’s Option B+ program, a woman is counseled and tested for HIV during regular antenatal care visits. If she tests positive, regardless of the CD4 count, she starts life-long anti-retroviral therapy (ART) [7]. Option B+ is an important initiative that safeguards both the mother and infant, but uptake remains limited despite advocacy efforts [8].

### HIV and PMTCT in Ethiopia

During the last two decades, remarkable improvement was recorded in demand creation, expansion of HIV testing, and expansion of PMTCT services in Ethiopia. This has resulted in a decline of HIV incidence in Ethiopia during the same time span [9]. However, the most recent Ethiopian Demographic and Health Survey (EDHS) data show adult prevalence in urban areas is at 3% (4.1% of women and 1.9% of men) [10]. The same report documented that knowledge about HIV transmission and prevention among those aged 15–49 years remains limited, at 38% for men and just 20% for women. In addition, most Ethiopians do not know their HIV status. Only 40% of women and 43% of men have been tested for HIV and received their results, with variations between regions and residence [10]. The highest regional HIV prevalence is in the Gambella region in the western part of the country, estimated at 5.7% when this study was conducted [10].

Following WHO’s recommendation and global commitment to end HIV/AIDS, Ethiopia implemented Option B+ beginning in August 2013 with the objective of reducing new HIV infections among children [11]. Studies show that following investment in demand-creation for PMTCT, the percentage of women ages 15–49 who knew that enrolment in PMTCT services could reduce the risk of vertical transmission increased from 10% in 2010 to 51% in 2016 [10]. However, studies have also shown that adherence to Option B+ services is compromised by number of ANC visits, place of residence, and male partner support to pregnant women [12, 13].

### Men’s involvement in PMTCT in Ethiopia

Lack of male partner involvement in maternal health has long been recognized; increasing male engagement as a means of improving maternal and child health has been a goal of the United Nations, nation states, and non-governmental organizations [14, 15]. Male partner involvement in antenatal care (ANC) and PMTCT is generally low in Ethiopia. Adherence to treatment and considerations of PMTCT service use is affected by a lack of support from male partners during pregnancy and childbirth [12, 13]. Ethiopian men tend to prefer their traditional role as breadwinners to involvement in maternal health issues, a domain men see as the sole responsibility of women [16]. One study showed that many men in Southern Ethiopia do not participate in birth preparedness, including ANC attendance with their partner (45%), saving money for an emergency or the delivery (30%), or making a postpartum plan (75%) [17]. A meta-analysis of studies in low- and middle-income countries indicated that almost half of male partners accompanied their wife/partner to ANC, with rates in Ethiopia ranging from 9.9–88.5% [18], with men in urban settings accompanying female partners to ANC service and institutional delivery [19, 20].

One small study in Gambella, specifically, found that 84.6% of men in the Agnuak zone planned to attend at least four of their pregnant partner’s ANC visits (although actual attendance was not measured), and only 7.9% of women surveyed indicated they made decisions regarding obstretric care by themselves [21]. A focus group study conducted in the same region showed that not delivering at a health facility was associated with the male partner making the decision regarding place of delivery [22]. According to the EDHS, 27.3% of pregnant women had not received ANC in Gambella [10].

### Men’s involvement in PMTCT in Africa

Evidence shows that a lack of support from husbands or male partners is a significant barrier to the uptake of ANC and HIV testing services among African women more generally. Women are reluctant to accept HIV testing and adhere to treatment during pregnancy if their partners do not have a stake in such programs [23–26]. Involvement of male partners in PMTCT service roll out, especially in low resource and high HIV-burden settings, plays a useful role [27]. Yet, existing initiatives to involve men in PMTCT interventions treat men as facilitators and tools of PMTCT program uptake rather than key program targets [24]. A systematic review of male involvement in PMTCT of HIV in sub-Saharan Africa revealed that women are the primary target of antenatal interventions. As a result of this lopsided approach, interventions neglect males as contributors to preventing HIV transmission from positive mothers to their fetuses. The review further showed that males do not see themselves as part of the equation; men consider pregnancy and associated health care as solely within women’s purview [25]. Other studies found that male partner involvement in PMTCT contributed significantly to a reduction in infant HIV transmission [26, 28].

Unfortunately, there is little evidence that explains men’s role (if any) in the prevention of vertical transmission, especially in settings with higher HIV prevalence. This study aimed to explore the role of men in preventing perinatal HIV transmission in the Gambella region of Ethiopia. More specifically, the study aimed to understand the perceived role of men in efforts to mitigate vertical transmission.

## Methods

### Ethics statement

The Johns Hopkins Bloomberg School of Public Health and the School of Public Health at Addis Ababa University reviewed and provided ethical clearance for the study. Permission and a letter of support were obtained from the Gambella Regional Health Bureau, which facilitated entry into the community and engagement with health care workers at the health center level.

Written informed consent was obtained from all participants. Researchers explained the purpose of this study and use of its results, and participants were assured of their anonymity during the consent process. Researchers informed every participant that the study’s outcome would not directly benefit them but could potentially provide useful information for PMTCT programs, benefiting both mothers and children. All participants were told that the information they provided would remain confidential. Accordingly, quotes were organized without identifiers.

### Study setting

Gambella is one of the nine regional states of Ethiopia located at the western tip. It borders South Sudan to the west and southwest, the Southern Nations, Nationalities and Peoples’ Region (SNNPR) to the southeast, and the Oromia Region to the east. The regional population was estimated at 409,000 in 2015, including around 270,000 South Sudanese refugees [29]. This study targets the two largest ethnic groups in Gambella: the Anuak and Nuer ethnic groups. According to the most recent national census (2007), the Nuer constitute about 65% and Anuak 30% of the total population of Gambella. The Anuak sustain on crop production, fishing, and hunting; the Nuer are mainly pastoralists dependent on livestock rearing. The most recent population-based HIV impact assessments documented that Gambella had the highest HIV prevalence in Ethiopia (5.7%) when the study was conducted [30].

### Study participants

Participants were married women and men and pregnant women who attended a health center during their recent pregnancy in the selected weredas and health centers of the Anuak and Nuer zones. The Anuak and Nuer are the two most prominent ethnic groups in Gambella.

Women who participated in the study were either pregnant or had given birth to a child no more than a year prior and attended ANC during their current pregnancy or last delivery. Furthermore, they were healthy and willing to participate in the study. Male participants were from the villages within the catchment of the health center where women received ANC services. They had partners who were either currently pregnant or who had delivered within a year of the study. The men were eligible to participate irrespective of whether their wives participated in ANC. Key informants included local opinion leaders, health extension workers (HEWs), and maternal health and HIV focal persons at the facility level. They were identified based on their recognized positions within the communities and health facilities.

### Method of data collection

Data were collected using in-depth interviews (IDIs), focus group discussions (FGDs), and key informant interviews (KIIs). The tools were translated from English to local languages by native speakers and back-translated into English by other natives. Two research assistants with language fluency (Nuer or Anuak) were recruited and trained for two days on the objectives of the study, data collection procedures, and ethical treatment of human subjects before commencing data collection, which ran from August to September 2018.

Researchers completed ten IDIs (five in Nuer and five in Anuak) with eligible women. Researchers held four FGD sessions with a total of 63 husbands of women who were either currently pregnant or had delivered within the previous year. The FGDs took place in villages within the catchment areas of the two health centers. Researchers conducted KIIs with opinion leaders in the respective study settings, maternal health focal persons at health facilities, and zonal and regional health bureaus. The FGDs and KIIs generated information on a range of topics including local beliefs on visits to health facilities by women for ANC; awareness about HIV, transmission, and prevention; HIV counselling and testing in connection to ANC and PMTCT; as well as the role of men as husbands in the health care of women during pregnancy and delivery. Interviews and focus groups were conducted with participants in each category until saturation of themes was achieved.

### Data analysis

Each day, researchers compiled field notes for all data collection activities conducted. Audio-recorded materials were transcribed verbatim and translated to English. Both transcripts and expanded field notes were read and re-read, and two independent reviewers developed inductive codes. Researchers categorized the codes based on the research questions and meanings to ensure consistent understanding. Thematic analysis using inductive coding [25] was employed to pull the findings together, triangulate by participants and methods, and interpret without deviating from the meanings participants intended. MAXQDA version 11 was used to facilitate data coding and organization. Verbatim quotes were chosen to illustrate findings shared by participants (see S1 Table).

### Data quality assurance

Research assistants were trained on the study’s objectives; method of data collection; and on consenting, recording, transcription, and field note procedures. During data collection, daily debriefings were held to improve questions and probing. One of the investigators (MK), who stayed in the field with the data collectors to facilitate daily debriefings, randomly checked the expanded field notes and later a sample of transcripts against respective recordings to ensure transcription accuracy.

Following the initial data analysis, the findings were triangulated by source of data and data collection sites and shared with focal persons in the health sector to validate the findings. Although researchers believe sharing results with participants at the community level is important, this level of data confirmation and results dissemination was not possible due to financial constraints; given the level of infrastructure and low literacy in the communities, this would need to be done in person.

## Results

### Socio-demographic profile of participants

A total of seventy-nine people from Anuak and Nuer communities participated in the study. Four focus groups were conducted in each of the respective communities. A total of 9 key informants representing two opinion leaders each from the two communities and those in the health sectors at wereda, zonal, and regional levels participated in interviews. In addition, 10 in-depth interviews with women attending ANC were completed. All community member participants were married and reported having at least three children. Opinion leaders, men, and women participants at the community level were all subsistence farmers and reported following the Protestant religion.

### Men’s roles as providers and protectors

Male and female participants indicated that men are responsible for providing basic support—such as food, housing, and clothing—for all members of the family, particularly his wife and the newborn baby. In addition, husbands are expected to protect the family from external harm, such as raids from other ethnic groups within Gambella and beyond.

A husband is also expected to provide money to his wife should she want to visit a clinic about a health problem. Participants reported that the husband is not expected to object when the wife requests money for health services. In the study communities, a husband demonstrates his success by fulfilling his family’s basic needs. As one participant stated,

*A weak husband who fails to fulfil his responsibility of providing enough food for family members, who does not have an acceptable house, who does not buy clothes for family members, and fails to offer care and love to his wife during pregnancy and after delivery, is not respected by the community members. As a result, men are expected to work hard to meet his family’s expectation*

(Woman, IDI, Dipatch).

### Male gender norms

While the community holds men accountable for protecting the health and well-being of the family, accompanying or encouraging wives to visit health facilities is not expected. Men’s role regarding women’s attendance at health facilities for ANC or HIV services is limited to providing their wives with money. Women do not expect their husbands to accompany them to health facilities, even in urban settings. One of the women participants argued,

*Husbands have several roles, and in our society, women are responsible for their own health. The husband, however, is expected to give her money*

(Woman, IDI, Krungeng).

### Purpose of ANC

Research participants commonly argued that there is a gross lack of information on the purpose of ANC as a service to pregnant women. All participants argued that women are expected to carry pregnancy to terms without much support from a health facility. One of the participants clarified that,

*There is no such thing as visiting a health facility by women in connection to pregnancy as we know. This has become a fashion only recently for reasons we do not know*

(Man FGD, Krungeng).

Two out of three men explained that they do not know the value of ANC. One of the participants stated,

*Neither we nor our wives know the purpose of ANC services at a health facility in connection to pregnancy*

(Man, FGD, Dipatch).

### Link between ANC and HIV services

Participants did not know how ANC and HIV counselling and testing (HCT) services were linked. Although health professionals and HEWs could explain that ANC is an entry to HCT services at health facilities, both men and women participants did not understand the linkage between ANC and HCT. A participant from Krungeng stated,

*ANC service is not known much in our area. Women in our area carry pregnancy to term with no problem and without any check. With HIV widespread in Gambella, women’s visit to facility in connection to pregnancy is being pushed by health professionals*

(Man, FGD, Krungeng).

Another participant stated,

*Attendance to health facility during pregnancy is useful since if the health of a mother and fetus are protected and the mother could be given medicine to protect their health*

(Man, FGD, Krungeng).

Similarly, another participant stated,

*During pregnancy, women visit health facility to check if they have HIV and to start taking medicine if the woman has it to prevent the fetus from getting HIV before the problem gets serious*

(Man, FGD, Dipatch).

### ANC follow up is women’s role

While participants commonly argued that pregnant women are not expected to visit a health facility during pregnancy unless they are sick, there was no objection by husbands should a wife want to do so. Participants emphasized that the woman’s interest in connection to her decision to visit a health facility is respected, and husbands are expected to comply with financial means. One of the participants stated,

*Although pregnancy is normal and there is no need to visit a health facility in connection to it, men in this area do not stop wives from visiting the facility*

(Man, FGD, Krungeng).

Both women and men participants, and health professionals, emphasized that local culture both in Anuak and Nuer does not expect men to accompany or encourage wives to visit a health facility. Accompanying wives to a health facility is not part of the role of men, while men are expected to provide money that wife may need for a visit. Participants’ dominant view was that men do not object to wives visiting a health facility nor do they resist giving money. However, participants from the health sector argued that more recently relatively educated husbands and those in urban settings have started encouraging their wives to visit the health facility in connection to pregnancy, although accompanying them on their visits is still uncommon. One of the participants stated,

*Husband accompanying wife to health facility is not usual and is not expected of men. In towns some men encourage their pregnant wife to visit in addition to giving her money. The health sector’s consistent awareness creation effort, particularly in urban settings, may have contributed to this*

(Health professional, KII, Gambella).

### Emerging stigma in connection to health facility visits for ANC

Findings revealed that ANC service is a relatively new development in the region and is widely associated with the spread of HIV. There was a common perception by men and women alike that visits to a health facility during pregnancy are associated with prevention of HIV for the fetus. Female research participants indicated that visiting a health facility for ANC services when pregnant is considered shameful since it is associated with HIV status. One of the participants stated,

*During pregnancy, it is normal that women feel tired, hate smells, and may even vomit. This is not considered as a problem. So, a woman does not visit health facility for these and husband is not expected to do anything*.

(Woman, IDI, Dipatch).

Another woman argued,

*Visit to health facility during pregnancy helps to prevent the fetus from getting the virus*

(Woman, IDI, Krungeng).

Male participants explained that there are efforts to bring men to health facilities along with their wives. However, in as much as local culture does not expect men to accompany their wife, a majority of the men expressed fear of being forced to test for HIV if they did go. One of the participants stated,

*It is discomforting to visit the health facility along with a wife. This is not normal here. In addition, we fear forced testing for HIV at facility*

(Man, FGD, Gambella).

## Discussion

This study found that local culture in the Gambella region of Ethiopia does not encourage wives to visit a health facility in connection with a pregnancy let alone men to accompany them on such visits. In Ethiopia, evidence from multiple cultures reveals that men are not expected to be part of maternal health care, including PMTCT service delivery. As a cultural practice, men are held accountable by the family as well as the community to be responsible for providing basic needs such as housing, clothing, and food for family members and to represent the family at communal forums [31, 32]. Seeking maternal health including ANC, HCT, and PMTCT are considered the responsibility of women. This is not unique to Gambella. In sub-Saharan Africa, where maternal health problems, including HIV, are long-standing, socio-culturally constructed gender roles distance men from women’s health care [33, 34]. However, men in Gambella are responsible for and expected to pay for all health service needs of their wives, whether the women feel sick or intend to visit a facility in connection to pregnancy.

From these findings it is clear that visits to health facilities by women and in connection to pregnancy is a recent phenomenon in Gambella aligned with the spread of HIV. The lack of understanding of the value of ANC is conflated within the community with adult HIV issues. Traditionally, the community believes that pregnancy is natural and that women should be able to complete it without visiting health facilities, a belief that can affect the level of ANC service use [32]. However, the consistent awareness-creation activities and encouragement of husbands to accompany their wives to such services appears to have negatively affected the outcome. In Gambella, belief about association between ANC services and HIV status was found to be widespread and stigmatizing. Men expressed fear that accompanying a wife may lead to forced HIV testing for him. There are similar other studies showing that the association of ANC visits with a positive HIV test may be linked to a fear of divorce among women since husbands often attribute a positive HIV status to a moral failing on the part of the woman [16, 35].

Lack of awareness about the links between ANC, HCT, and PMTCT, and how all of these services could benefit an infant reinforces the cultural domain of gender roles. Furthermore, fear of testing positive not only discourages male involvement but also challenges expanding ANC and HCT services utilization. Despite evidence suggesting the need for male engagement to improve maternal health indicators, there are persistent barriers [23, 25, 28]. This study implies the need for well-organized and socio-culturally sound interventions to modify gender roles, thereby building values that accommodate men’s role in routine health care for pregnant women. Available evidence shows that ensuring men’s engagement in women’s health requires interventions at the health facilities, in the community, and in educational and economic programs to achieve sustainable health interventions [34, 36].

## Limitations

This study generated valuable evidence suggesting reasons for the lack of male involvement in PMTCT service provision in the Gambella region, particularly for the Anuak and Nuer ethnic groups. There are limitations to this research, however. Although daily debriefing helped the researchers gain insight on the narratives shared by participants and to improve the interviews as they were conducted, doing research in a community where language barriers exist may have limited the depth of the narratives expressed. To mitigate this, data collectors were local researchers fluent in the local languages. Despite this, nuanced meanings of the narratives provided could have been lost during the transcription and English translation processes. This study was also limited in that findings are focused in more rural areas of Gambella. As is the case with any qualitative study, findings are not generalizable beyond the included participants. Future studies should address any differences in opinions regarding male involvement in antenatal care and PMTCT in urban settings of Ethiopia. Finally, power dynamics between the researchers and the local community members may have influenced the candidness of participants in expressing their true opinions. However, consistency of findings across data collection activities, including across FGDs, suggests that participants were truthful in stating their opinions despite the research study context.

## Conclusion

Male engagement in women’s reproductive health is crucial beyond their role as financial providers. In the Gambella region of Ethiopia, communities need a better understanding of the links between maternal and child health and ANC during pregnancy, the benefits of health facility usage beyond addressing symptoms of illness, and the benefit of male involvement for the health and well-being of the family and community at large.

## Supporting information

S1 Table Data by themes and respondents. (DOCX)
