# Supplementary material for: The role of men in antenatal care and preventing HIV transmission from mothers to infants in Gambella region, Ethiopia
Source: PLOS Glob Public Health. 2022 Aug 29;2(8):e0000879. doi: 10.1371/journal.pgph.0000879 (PMC10021612; doi:10.1371/journal.pgph.0000879)
Supplement: S1 Table — (DOCX) [file pgph.0000879.s001.docx]

**Supplementary File 1.** Data by Themes and Respondents

| **Themes** | **Health Professionals** | **HEWs** | **IDIs/women** | **FGDs/men** |
| --- | --- | --- | --- | --- |
| Explaining pregnancy |  | - Appreciate the pregnancy since it is important to life continue - Pregnancy is normal after marriage - It is important part in life | - I appreciate the pregnancy because I know the benefit of the child - respect community has for a women who get pregnant - Getting pregnant is blessing – husband is happy, my family is happy and the community is happy - Women gets pregnant when she is healthy and happy - Husband and wife are happy when pregnancy occurs - Everyone in the community is happy since this is an addition to the Nuer - Pregnancy is source of happiness for women and the husband - Everybody wants another child for the society - I appreciate the pregnancy women b/c it’s the new child | - Not only family members but also members of the local community are happy when a woman got pregnancy - Community appreciates woman for bearing new child - Getting new child is good sign for the family - Children are wealth – more children is considered valuable - New child is an addition to the Nuer - I am very happy when my wife get pregnant. - Pregnancy is the most important moment of happiness for all – husband, community and family - It is an addition to the Nuer - when my wife get pregnant I feel happy and lucky – since this is a contribution to Nuer - We need more and we are happy as a community - Because the fetus was belong to us, the nuer |
| Manifest – Moment of happiness, new child is revered, family’s contribution to the community and the Nuer, every marriage is expected to produce child, more children ‘important’ ‘wealth’ ‘valuable’  Latent - Women feel responsible and feel accountable to make husband, family and community happy. Women under deep pressure to bear children. Bearing new child is a relief and source of happiness for wife and husband for public expectation is fulfilled. More child is always better  More latent - Competition among those who are fecund and distress among those who do not get pregnant. Failure meeting responsibility is not appreciate by the community | | | | |
| Problems in connection to pregnancy | - Community considers pregnancy as normal - There were no problems related to pregnancy - People do not see any problem during - Pregnant and delivery are considered normal - Shortage of blood could be a problem during delivery - Bleeding is critical problem during delivery - They get tired and discomforted during pregnancy - Shortage of blood during delivery - Access to services due to distance to facility are problems - Delayed visit to health facility during pregnancy | - Get tired, hate food, can’t do as much job - No problem in connection to pregnancy - No do not think there is any problem - Natural to hate food and be tired | - There is no special problem in connection to pregnancy - It is natural to be unhappy, hate food etc - no health problem in connection to pregnancy - This health problem for the pregnancy are not unique. Women feel pain, hate food and are tired - If healthy and happy I stay healthy during pregnancy – no problem - No health problem due to pregnancy - When pregnant women always get sick and this is not serios and no need to visit health facility - Women are not happy, feel weak, hate food during pregnancy – this is normal and not considered as health problem - Health problem for the pregnancy are not unique - feeling of pain, hate food and getting tired are normal | - Morning sickness - To me pregnancy time has several problem – not happy – comfort is not there, do not eat enough and feel tired - Women vomit, can not eat and not happy during pregnancy and this is not a problem – normal - Community know that unhappiness and not eating food during pregnancy is common and normal - There is no special problem during pregnancy. Some women get sick and visit health center which is also normal - Visit to health center is not known as we know. It became a fashion now. There is no reason why and we do not know the reason - My wife for example did not visit health facility during pregnancy. She was not ill and I do not know the use of it - My wife bore two children without visiting health center and there was no problem - Most educated or who listen to health the workers visit but most are not - Community do not believe and so do men that pregnancy has any problem - Pregnancy is considered normal - Problems related to pregnancy includes unhappiness especially if quarrel with husband and do not have as much love - Problems related to pregnancy are pain, bleeding before and after delivery - No problem during pregnancy and delivery. Sometimes labor is difficult but after child birth no problem. All relatives visit and it is ceremony - If husband fails to fulfill basic needs then it is a problem |
| Manifest meaning – hate food, feeling tiered, vomiting etc. These are normal manifestations of pregnancy and are not considered as problem.  When/if women feel sick [which is not normally the case], she has the right to go to health facility and husband has the obligation to provide money but is not expected to accompany  Latent meaning – Health problem in connection to pregnancy is not for every woman. Those who often visit health facility are suspected to have HIV especially if they are not educated. Others wise educated ones and those who live in towns are now a days visiting health facility during pregnancy for ‘vaccination’ purpose. Normally for three out five it is not a problem and symptoms of pregnancy are not taken seriously.  Health professionals – in maternal health department at facility and management level attribute health problems as an outcome of delays to visit facility, distance and believe consequences as fatal while the rest of participants including HEWs share common understanding that pregnancy related problems are not recognized by local culture | | | | |
| Most affected | - Women who have sexual affair with more than one man - Women whose husband who visit other women in town - Side effect of the medicine for the positive ones - Women who are HIV infected often complain to have health problem - HIV positive women are often affected by different health problems | - Women who have HIV are most affected - No women are more affected than those with HIV - Women who are in town and who has husband with town women - I do not think there are women who are more affected - May be husbands who visit town may cause the problem | - some women who were affected with vulnerable to HIV. This is due to sex with many men, sell sex also is a problem - Those women with multiple sexual partner - I get that infection from my husband - Women who get HIV get from their husband since men are promiscuous with multiple partners - In our community there are women who get HIV infection from sex with different men - Men marry many women by men which is the reason for HIV infection - The case of this infection was multi direction with man have many wife and women have many man for sex Pregnancy is not a problem in Nuer | - Those who have sexual affairs with many especially with ‘prostitutes’ - Those drink much - Women who stay with community and engaged in sex with many men - Other participants agree that sex with many is a problem - Women are vulnerable to HIV due to having multiple partners - Women in our community are vulnerable to HIV because they did not trust the condom to protect their life with diseases HIV - Women in this community are vulnerable to HIV due to multiple sexual partners - There are women who have many partners and they visit services. - Vulnerable women are those who have shortage of knowledge on condom - May be these women who have more man get the problem. May be the town women are more affected - Women in town are more affected - Yes there were some women in this community who are affected these are the ones who have sex with many men - Those who drinks much visit ‘prostitutes’ - Culture does not allow sex with many men but those break culture and have sex with many male - Women in towns are the ones who have problem and use service |
| Manifest – those who live in towns, have multiple partners , who drink and have sexual affairs with ‘prostitutes’ are most affected. As few as 2 out five mentioned that poor state of life – weak husband who does not cater for wife’s basic need and those who do not offer care and love for wife facilitate for the women to be affected. On the other hand health professionals not including HEWs argued that those who are using drugs are often affected due to side effects and associated problems  Latent – living in town, drinking alcohol, lack of awareness about the problems associated to pregnancy make women affected BUT the problem often is blamed onto men by women while men blame on women. | | | | |
| Care to women | - After delivery women gets care by husband - Hard work is common among women and pregnant women are not spared (fetching water and collecting fire wood are few) - Women visit health facility at will and often is for vaccination - HIV positive women gets services at dedicated corner in the facility - If particular providers to HIV positive ones are not there women are unhappy (worried for their secrets is not maintained if support is sought from other provider) - Husband avoid wife to do heavy work during pregnancy - Husband organize accommodation and wives have enough food - Through 1 to 5 network at kebele level women are supported for ANC and HCT services - Would have many problem when they get pregnancy - tired and discomfort - Health workers give education on pregnancy - good for health of women - Those women from rural areas miss many appointment due to lack of transportation - HIV positive women take their medicine and given biscuits to encourage them to use their HIV medication freely with out payment | - Husband provide food and care after delivery - Husband stop wife to do heavy work during pregnancy - Men would take care of their wives avoid hard work - Women visit health facility if she is sick | - Husband do not say not when the wife want to visit health facility - I do not go to health center without my husband’s permission - If I want to go to health center my husband do not object – he gives me money - I visit health center when I am sick. If not why do I go? - She may go to health center to deliver if she has problems - If there is no problem, there is no visit to health facility - I think man or women having too much sex is a factor - Better to stop heavy work which husband always want his wife to do the job even if she is pregnancy - But if women is sick husband pay many and her sister or sister of husband take her will travel with her to HC - Pregnant must visit health center to check if sick - Going to visit health center to know what was going on about the health of woman - Health worker told me and my friends about bringing husband to health facility - I am ok but there are women in town who go with many men and men who have many women - If the infection happen through woman husband should follow the treatment - The women who has HIV would stay with community and there is no problem | - Husband is expected to feed the wife during the delivery – otherwise she loose blood. - If my wife want to visit health facility, she can and I do not say no. But, normally men do not go with wife to health center - Normally if my wife is sick and interested to visit health facility during pregnancy, I help her with money and encourage her. But, man does not go to health facility and not expected in the culture - But, man does not go to health facility for this is not expected in the culture - If pregnant wife become sick she has the right to visit health facility - So, when wife is pregnant, all men are responsible to provide care to make her happy and healthy - Husbands prevent wife from hard work and make her to be happy when she is pregnant. - If the women is sick during pregnancy husband give money to visit health center - I care for my wife I want her to be healthy when she got pregnancy she must go to health facility to take care of these problems - When my wife was visiting the health facility for her pregnant I would agree to the mission of her to take drug for her and fetus because I would care about my wife - I would tell to my wife to go to health facility for ANC visit - I would go to health center if needed – if I am called. Otherwise I do not go with my wife. This is not the tradition to be with women - I talk to my wife when she get pregnancy to stop her to stay at my home without do hard work only to follow the immunization - If pregnant wife want to visit health center, husband do not say no – but give her money - Husband as owner of the wife is responsible to care for his wife ‘s health - Husband is responsible to pay for health care and provide food and cloth after delivery - Health professionals are responsible to care - My family also have their own responsibility to care for pregnant women help her when she can not do all job. |
| Manifest – Husband is responsible to make wife happy by buying her food and cloth after delivery while he encourage here to be engaged in heavy work during pregnancy. While health professionals argue of provision of care from local level to health facility level through network of women at village level and provision of dedicated service to pregnant women and those who have HIV, all participants argue that husband is responsible and is in fact culturally expected to provide support (provide money for health facility) and allow her to visit health facility should she wish to and ensure she has enough food and wear for herself and the new born after delivery. However  Latent – Husband is primarily responsible to care both during pregnancy and after delivery. Such responsibility does not include accompanying wife to health facility since Nuer husbands are not culturally expected to and are not interested to. As few as one in 10 participants pointed out that if the health facility calls them they would go with wife although this was not quite established reality | | | | |
| Role of men in women’s visit to HF for ANC, HCT, PMTCT | - Questions widely show that there is need for a man to go to HF with wife. Men say they are expected to be strong to feed the family and give money to wife for services - If women is infected there would be disagreement between them that things would prevent the men to visit the health center - Even if invited husband do not come to health facility. - Usually husband do not come - Men are expected to be active in economic arena - Responsible to keep family with food and all other basic needs - food for his wife and need good clothes for them and health of the family - Men have obligation to feed the family and wife during pregnancy after delivery to avoid bleeding - Allow wife to visit health facility if she wishes if she is sick and in connection to pregnancy - Husband in urban area advice their wives to visit HP for ANC to prevent the problem from happening - Normally Nuer men do not visit the health center with wife – this is not with his role – expectation - Husband do not even know why wife visit HF since this is not common - Men know from wife on the visit she made to health center - The wife must visit health facility with her husband to know the result of each other after HIV testing - The husband have little insurance to pregnancy in Nuer zone for the health of women - There was a man who do this support at Nyinenyang but majority do not do the support. The culture in Nuer do not support this - Husband do not know and the culture is not supporting - if the husband had money he would tell the wife to go alone or with a relative - Wife is responsible for care of children while husband care for the entire family - Health professional continuously advice families (women) to bring husband along | - If woman is sick when pregnant she can visit and husband do not say no - May be few men in urban area would accompany wife to health center to take test during pregnancy - Pregnant women can go to health center for her pregnancy and men has not other role but give her money - Few men in kuergeng community who support their wives to attend ANC and counseled tested against HIV - Men of Nuer do not go to HC with wife and culture do not support this - Husband is responsible to ensure the family have food to eat and cloth to wear - Men do not discourage wife from visiting health facility if she is interested - Husband gives money and wife arrange to visit health facility - The men or husband encourage their wives to complete their vaccination during pregnancy - In the community husband do not visit health facility with the wife but help wife to visit - Yes, there is no man who say no to wife to visit HF if she is interested - Giving money to wife is expected from the husband to visit health center - Community do not have role but husband should give money - Community do not appreciate if husband go with wife to health center - Men are responsible to keep their family and protect them from harm - Health facility teach husband through wife but they do not go | - My husband is not happy to go with me but the health worker invited and he came only once - My husband support me with food and clothes and that is husband’s role - My husband advised me to attend ANC and counseled tested against HIV - Support me with money and food no any other thin - Men in Gambella do not involve in women activities - whether the women is pregnant or not. Women are responsible for HH activities, fetch water and firewood and care for children. - My husband continuously stimulated me to visit health center during pregnancy - My husband help me - give me money to visit health center when there is problem during pregnancy and labor - My husband was play the role to ensure my life and the fetus by give money and advice me to visit health center when I have a problem - The opinion of the men to improve would tell their wife to visit health center when the wife get pregnant - My husband support the family with economy - There would be problem if the husband was not active economic problem occurs - Normally husband do not go HF but give money to wife if interested - The man would care for me economically and is expected to care for the family. If I am sick he gives me money for the health service - The man would ensure the wife when she get pregnancy for food. Normally the man support his wife – food/fish, cloth letter - If the man is nice he did not go with his wife b/c he didn’t see the health of woman as responsibility for him - Man in Nuer has many wife and do not follow every wife and support. But he give money, food and cloth. That is the role - Culture do not allow. Man do not want reference as women so his support is only with money - The men are providing food during the pregnancy and latter and should you would go to community and tell them to keep their women - Normally sister and sister of the husband help pregnant women when she visit health center. Man do not care taking the wife to health center - If the men has money he would not invest for the wife to visit health center. He would like to consume his money and consider wastage to visit the health center - The women and fetus would be safeguard by the men who have responsibility to upkeep their family - Going to health facility is not a must for man except the expectation to give her money - Its true men do not have idea on this. Women have better information - Yes the man were supply the more support their wives to tell them to visit health center if she has problem | - Some husband (few may be 2 or 3 out of ten) inspire their women to go to health facility for check up. Majority of husband provide money and do not object her visit to HF if she wants to - Men help their women by giving food and cloth when the women become pregnant - Before her delivery men make preparations (buy clothes and improve house) - During buying clothes and ensuring availability of food is key role - After delivery, making enough food – fish and hunt - Few men support their women to attend health center during pregnancy - Most do not care on visiting health center but do not object due to Nuer culture. - Men are responsible only for food and cloth - Normally if husband object wife’s visit to HF, she can complain this to her parents and husband will be in trouble - Involvement of males in women’s health in Gambella is difficult to improve. - Changing the practice of marrying more than one wife remains difficult since this is culturally ok - When my wife experienced symptoms of illness i used to encourage her to go the health facility to get support and give her money - Most women they don’t want to go to health facility because of fear or shame to see or examine by care providers - I didn't know the reason for pregnant women visiting health facility at services - Health care providers we heard teach on the benefit of visiting HF during pregnancy and delivery - Some of our community were getting that information from HEWS - I think most of us do not know about this - what I know that men have role to take care for their wife as well as for the fetus during pregnancy, delivery and after delivery by providing food, cloth and house - I help my wife not to do or carry heavy loads - Advice women not go far away and do heavy work - There were two to three men in this village who help their women when they get pregnant to attend that services. - Men are not doing indoor works because if they do other people take as shame to them - I am not supporting my wife due to lack of time for me - Men in Gambella do not care about maternal health care - I would tell to my wife to joint health center for her health - to get health care for her pregnancy - She may get checkups and husband do not say no. If problem is there parent of the wife will take measure. So, wife visit health center - At socio-cultural level there were social division of labor example, in nature work such as collection of woods, food processing, making cleanliness of indoor and outdoor, feeding children…are mostly taken as women duties. On the other hand work such as harvesting, house and fence building, hunting – everyone is expected to comply to such role - Men are inactive to support their wives due to polygamy the culture of the Nuer community. - Males of Gambella did not care about the life of women they were very weak from the health care of the women - When my wife visit the health facility I would be luck whether she visit without permission from me I like it. - There was no problem about her visiting hospital. I tell her go if she wants but I am not going with here because this is not male’s job - Most women they don’t want to go to health facility due the cultural influence and afraid of abortion in relation to drugs sometimes they will provide with - Normally woman go alone and husband make payment - I would make my lovely wife to feel happy when she get pregnancy and help he to go to health care facility. - I will go to health center with her to pay the cost which the medication need - During delivery I would need the clothes for new born and to stay at good place by improving the house – roof and wall to make wife happy and visitors also - In this community there are men supporting their women by encouraging them to go to health facility. This is not all men may be small (few) - The men will not have time due polygamy - The men did not have willing to visit health center and tested this was problem of males in Gambella |
| Manifest – Men are responsible to make food available to the family, maintain house before women deliver and buy cloth for the new born and the family. Accompanying wife to health facility is not among the role of men among the Nuer. However, husband is not supposed to forbid wife from visiting health should she wants to. While a third of health professionals who participated in the study share same opinion as others, the rest felt husband’s role is to accompany wife to ANC and get tested for HIV. One out of five participants generally argue that husbands in towns and educated ones at least encourage wife to visit health facility while the remaining proportion do not even ask what happened except when told by wife.  Latent - Husband is expected to keep the family economically well – food, cloth and money needed for health services. First community do not hold men accountable to accompany wife to check her pregnancy (visiting facility for mere pregnancy is not considered normal) and secondly husband has several wife that he caters only for their economic need which is basic expectation while health care (when sick and/or for pregnancy related check ups) is the responsibility of each women. So, accompanying women to health facility is not culturally defined as men’s role. Educated husbands (as many as 1 out 5) argued encourage or ‘may’ accompany them.  With HIV there is fear that visiting HF will break bad news about status that even if invited by health professionals through the wife husband’s find it difficult to turn up to health facility  Me – while there is new developments where one in five husbands who are educated and in town encourage wife to visit health facility and ‘may’ also accompany, this is not clear yet if husband’s are convinced and see value in such visit or are called upon by wife.  Male accompanying  Wife to HF  Before HIV is known to now | | | | |
| Barriers to men’s accompanying wife to HF | - There is no information about this [PMTCT, Option B+ and ART] to the community - People do not know about HIV as serious disease –consider it as any other disease | - I think no knowledge and do not know about this | - My husband does not know if going to health facility is necessary during pregnancy - When the men are not active for the health of the women in connection to ANC, HCT and Option B+, I think the problem is lack of understanding | - Other participants – lack of information and tradition. - We do not know details of this. - Barriers were lack of commitment from the men to care for their women due to lack of informaiton. Health center people should give education to all - Male and women alike do not know about ANC,HCT and Option B+ - What I know about attend I have no more knowledge - At individual level men are not active to support their wives due to lack of knowledge - at social level men are not support their wives due to social division of work i.e. indoor work is for women but not for men - Lack of knowledge to care for their wives. What can I do – do not know this |
| Manifest – Do not know the importance of the visit and understand the value of accompanying women to health facility. Visit to health facility accompanying wife is not known by the community and is not appreciated.  Latent – men’s role doesn’t include accompanying wife to health facility. This implies that even if husbands are made aware of the value of accompanying wife to health facility, they may not do that since there is no cultural pressure and secondly the fact that visit to HF is connected to HIV, now fear and such visit/accompany is associated to HIV will make it difficult to make husbands accompany wife | | | | |
| Suggestions to improve men’s role | - teaching of health facility - ore education to man is very imporatnat - Government work on capacity of men |  | - I would wish this topic will be better to teach the males to involve in this program - I feel more education is needed to the public and men - Education to community to change tradition | - Our idea is the regional health bureau to prepare training about this topic - Education is important. Our tradition is still good and we must know how to change - Teaching the community and man to appreciate the women for that responsibility to care of their women healthy - The organization should be create the training to aware the males to know the care taken of the pregnancy - t would be beneficial if enough awareness education is given to men to engage of this program - in addition there should be community awareness at the community level in their villages. Also there should be trained facilitators to facilitate the sessions during group education - The regional health bureau should be prepare the programmed of training for male awareness to care for their women - The community provided with the information about the role of men in supporting their women |
| Manifest – Awareness creation, knowledge building came out immediately as solutions. While health professionals and women participants suggested teaching men as a solution to improve men participation, men FGD participants and again women participants suggested community level teaching by health sector.  Latent - | | | | |
| Visit to HF | - HCT during pregnancy assistances the child to prevent from getting infection because the mother can use this services - ANC visit is useful for HCT during pregnancy women b/c the ART, PMTCT option B+ were the helpful when the women get positive HIV |  | - HCT during pregnancy was preventive the infection because action is available |  |

| Value of PMTCT, HCT, ANC | - Most women who go to HCF are women who have awareness , But the other are not attending due to lack awareness | - Pregnant mothers come to health facility because they are told by health workers at community level to get care to be healthy | - The benefit of the services are to find and treat the problem before it is serious | - She visit health facility when she feels sick (KII-CL) |
| --- | --- | --- | --- | --- |
|  | - The benefit of those services are to keep the health of the mother and the fetus. | - most do not come to facility due to long distance travel to HF | - I got information from health care providers through education during facility visits for medical check or during pregnancy | - HCT during pregnancy helps to prevent the fetus from getting infection because the mother may linked to services earlier (KII-CL) |
|  | - HCT during pregnancy helps to prevent the fetus from getting HIV infection because if the mother test result shows HIV positive she may linked to PMTCT Option B+ service earlier that can protect the fetus from getting the infection | - Men as husband know about the services through their health care providers at health center and through group education sessions at community level | - Yes HCT during pregnancy helps to prevent the fetus from getting infection because the mother may linked to other services earlier | - Visit to health facility during pregnancy is useful. Health professionals will test the women for HIV and give her medicine if she is positive. This helps the fetus to be free from HIV (KII-CL) |
|  | - ANC visit is useful for HCT during pregnancy because all pregnant women are subjected to test for HIV during the visits and start PMTCT option B+ if the reported as positive for HIV | - HCT during pregnancy helps to prevent the fetus from getting infection because the mother may linked to other services earlier | - Yes HCT during pregnancy helps to prevent the fetus from getting infection because the mother may get support | - can say the fetus may not got infection if the mother got treatment earlier from health facility. And also if the newborn is cared during and after birth. |
|  | - the fetus or child may get infection before delivery if the mother didn't attend the services | - ANC visit is useful for HCT during pregnancy because all pregnant women are subjected to test for HIV during the visits and start PMTCT option B+ if the reported as positive for HIV | Yes ANC visit is useful for HCT during pregnancy because all pregnant women are subjected to test for HIV during the visits. | - think it will better if the woman go to facility to be checked for HIV infection before delivery |
|  | - HCT during pregnancy helps to prevent the fetus from getting infection because the mother may linked to other services earlier | - Seven out of ten they used to come and attend the services here in our HC | If the mother didn't take treatment, the fetus may get infection from the mother before delivery | - If woman know her status during pregnancy and if she take medicaiton on time that is good to protect the child |
|  | - Yes ANC visit is useful for HCT during pregnancy because all pregnant women are subjected to test for HIV during the visits and start PMTCT option B+ if the reported as positive for HIV |  | Yes HCT during pregnancy helps to prevent the fetus from getting infection since mother will get treatment | - In general( ANC is good opportunity for HCT because it makes the women to be tested for HIV and this is helpful for the health of the mother and his fetus). |
|  | - Two out of ten women in this community use PMTCT service in our HC to protect the new born from HIV |  | - pregnant women who attend ANC are subjected to test for HIV during the visit | - to know their status whether they are normal or abnormal and if they are abnormal just to start getting PMTCT option B+ before delivery |
|  | - PMTCT is a program which is working on those of women who are HIV positive to be protected from infection |  | - HCT during pregnancy helps to prevent the fetus from getting infection |  |
|  |  |  | - together with my husband should be tested to know our status | - This program helps to protect the fetus not to acquire infection from the mother |
|  |  |  | - Children are wealth. Those with many children are respected | - I will tell her to go to health facility to test for HIV and if we are fine is good but if we are not fine I will tell her to start PMTCT option B+. |
|  |  |  | - I do not know how transmission happens | - she will become healthy or happy if she got care when she encounter problems during pregnancy |
|  |  |  | - HCT is important to prevent the fetus from getting infection | - Mother who have HIV in her blood she must take continuously PMTCT option B+ service since this will help avoid infeciton of the fetus. |
|  |  |  | - There is no difference between women on the use of services in order to protect their and their fetus health | - I heard about HIV that there is a way that health care providers used to protect the child not to get the infection (meaning by giving the PMTCT option B+ drugs |
|  |  |  | - HCT during pregnancy helps to prevent the fetus from getting infection | - Those mothers who didn’t visit the health care facility, they can transmit the infection to their fetus |
|  |  |  | - ANC visit is useful for HCT during pregnancy | - If there is a woman who didn't come to health facility and also who have feeling of shame to visit health facility she can pass on the infection to her fetus |
|  |  |  | - Pregnant women should be tested for HIV to protect their fetus because men mostly are not willing to be test for HIV. | - The fetus may not get virus from mother to the fetus if she screened as HIV + and start PMTCT Option B+ earlier and also if she is well oriented by health care provider about how to take or use the drugs without interruption. |
|  |  |  |  | - ANC is good opportunity for HCT because it lets the women to be tested for HIV and start treatment earlier if they positive for HIV. |
|  |  |  |  | - For both husband and wife it will be good if they tested together |
|  |  |  |  | - When she screened as a HIV positive after she has provided with drugs, the health care provider will advice her that she must not stop taking this drugs |
|  |  |  |  | - In this community there are men supporting their women by encouraging them to go to health facility during pregnancy |
|  |  |  |  | - If the woman become pregnant better to tell her to go to HF for pregnancy ANC follow up |
|  |  |  |  | - When a woman become pregnant if she didn't follow ANC and other like HCT during birth she will face problem of anemia & others |
|  |  |  |  | - Problems which occur after getting pregnant up to delivery cause a big problem in labor time if the lady didn't attend pregnancy visits and also if she didn't take medication well she will be unhealthy even her fetus |
|  |  |  |  | - at last stage of pregnancy when she start to feel pain she will decide by herself to come to health facility for checking and treatment and she will feel healthy after treatmen |
|  |  |  |  | - This is true all people understand that it helps the prevention of fetus from infection |
|  |  |  |  | - To ensure whether the mother is healthy one part of activity is testing for HIV |
|  |  |  |  | - It will be good if both husband and wife get tested because it will be benefited for our children and for us also. |
|  |  |  |  | - ANC follow-up given to a mother at facility since it is use full, I used to encourage my wife to go to health facility to get ANC service |
|  |  |  |  | - Testing the mother only will not protect the fetus from acquiring HIV without starting the treatment |
|  |  |  |  | - During pregnancy It will be good if the mother tested for HIV because it will be benefited herself as well as for her fetus incase if her test result showed HIV+ |
|  |  |  |  | - If her test result showed HIV+ immediately she will put on drugs. And also testing male partner is good for his wife because he will start his treatment soon. |
| Manifest – while health professionals explained the value of ANC attendance as this links ANC attendees with🡪HCT🡪 and those who tested positive could be linked with PMTCT option B+ service while those who tested negative will benefit from ANC follow-up. They pointed out that two out of ten women benefit from ANC services. They further pointed out that the service (PMTCT Option B+) protects the fetus from getting HIV from HIV positive mother.  HEWs , women and men participants generally feel visit to HF during pregnancy is to get test for HIV and protect the child from infection.  Latent – ANC is linked to test for HIV and prevent HIV infection of the fetus before birth. Although there is growing understanding that visit to health facility during pregnancy is associated with HIV, there appears to neglect of ANC in its own right. So, there is misconception of associating visit to health facility during pregnancy to HIV. Those who visit health facility itself were found to have health problem, which in this case is HIV.  Me – Such misunderstanding pose challenge to ANC for in the long run due to imminent stigma (as many as three in ten women and four in ten men those visit HF has problem) – which is contrary to stay at home and deliver which has been known so long. | | | | |

| What is known about transmission of HIV | - Mother with HIV in her blood if she become pregnant and didn't attend ANC and didn't started treatment earlier can pass the virus to her fetus before birth. during birth and after birth | - the fetus may get infection if the mother didn't start the PMTCT option B+. | - If the mother attend facility visits and got services well, the fetus may not get infection from the mother | - the fetus may get infection if the mother didn't go to health care facility |
| --- | --- | --- | --- | --- |
|  | - fetus or child may get infection before delivery if the mother didn't attend the services | - after delivery if the mother didn't attend postnatal care for the child | - If the mother didn't attend the services and take the medicines correctly the fetus can get HIV infection from the mother | - the fetus may not get infection if the mother was attending the ANC and PMTCT OPTION b+ at HC or Hosp |
|  | - during delivery if the mother delivered some ware outside the facility |  | - I don't know but sometimes I heard that if the mother didn't take treatment the fetus may get infection from the mother | - when the child grown up he/she should stop breastfeeding because he/she may get the infection from the mother through milk or through blood contact during feeding breast when he/she bite the nipple and cause bleeding |
|  | - If positive women follow PMTCT Option B+, even if she feed her child through breast feeding, the child may not get the infection |  | - After delivery the fetus may get infection through breastfeeding(Milk) | - The woman should deliver at health facility rather than deliver at home because the child may get infection via unclean materials that can transmit the infection to the newborn |
|  |  |  | - if the mother didn't tested for HIV and start treatment, the fetus may get infection from the mother before delivery | - I will push her to deliver at health facility if this done the new born will be healthy |
|  |  |  | - After delivery the fetus may get infection through breastfeeding | - What I learned from this question is when the woman become pregnant she must be told to go to health facility to checked for her health status and during this time if she become positive for HIV |
|  |  |  | - I think if you do not know your health problem the child may be affected | - During delivery she will be advised to deliver at facility to be care for the new born not to be infected. |
|  |  |  | - I don't know |  |
|  |  |  | - Another is when the mother didn't go to facility to get ANC, HCT and PMTCT Option B+ services | - Fetus become infected when the mother born at home through using of unsterilized materials |
|  |  |  |  | - knew during health education is that the fetus may got infection from mother after birth through breast feeding if the child feed breast milk up to more than a year |
|  |  |  |  | - If the child soon after delivery give to other woman and feed that woman breast milk he/she may get infection in that way |
|  |  |  |  | - The fetus may not get the infection before birth but may get the infection after birth and there will be a care for this case |
|  |  |  |  | - Depend on what I have been aware if the fetus stop breastfeeding after 1 &1/2 year will not get infection from mother. |
|  |  |  |  | - The fetus may not get infection (virus) when still in the mother uterus because he or she have no blood contact from the mother |
|  |  |  |  | - may get after birth managed outside the health facility if the cord of the fetus was cut with un clean object(contaminated object with virus) |
|  |  |  |  | - After birth the child may not get infection if he/she got treatment with drugs |
|  |  |  |  | - Yes the fetus may got HIV infection from mother through breast milk |
|  |  |  |  | - f the mother give birth outside the health care facility easily she may pass on to her new born child |
|  |  |  |  | - he fetus may get infection (virus) if the mother didn't start treatment earlier |
| Manifest – Health professionals explained that if woman fail to attend ANC during pregnancy the fetus may get HIV. HEWs indicated that women should deliver in health facility and follow postnatal care so as to ensure new born is free from HIV. Delivery in health facility helps prevent HIV transmission from positive mother to the fetus.  Women participants explained that there is limited information about how and when HIV transmits but generally argue that if the mother didn't take treatment the fetus may get infection from the mother.  Men participants argued that visit to health facility helps since women will get education and medicines. Three out of five participants argued that breast feeding the source of transmission  Latent – one in five health professionals and none of HEWs explained HIV transmission during pregnancy, delivery and breast feeding. There was general argument that attendance of health facility is useful to prevent HIV infection from the fetus.  Me – Participants do not have clear idea on when exactly and how HIV transmits from positive mother to the fetus. Surprisingly even health professionals (although half of them were at management level did not explain modes of HIV transmission from positive mother to the fetus) | | | | |
|  |  |  |  |  |
| Awareness about PMTCT | - I think these services is not known by community |  | - I knew about the above services after I visited the health facility | - Most pregnant women choose not to come to health facility just to check pregnancy because of lack of awareness (CL) |
|  | - Many members of the community don’t know about HCT, PMTCT, Option B+. Who know about these may know through their wife and through adherence supporters |  | - Women are not coming to facility for visit because of lack of knowledge or problem of understanding about the importance of the services they will get from the facility | - we know there are women who are using PMTCT Option B+ here in our community (CL) |
|  |  |  | - Women including me do not know much. We heard about this from health personnel at health facility | - if she attending the visit new disease called HIV if got in her blood automatically she will put on the drugs not to transmit the infection to her fetus in the uterus. lastly the fetus will be born free from HIV |
|  |  |  | - heard but I don't know them | - What I know about the new disease known as HIV. If my wife got pregnant what I am going to tell her is to visit health facility. This time I don't know whether she do have or no HIV infection but if she become positive she will be provided with drugs that can protect from getting HIV |
|  |  |  | - Yes men knows about the above services. And they heard the information from health personnel at health facility | - In most case what I learn from this question if my wife become pregnant I will let her to go to health care facility to be checked but until this time I don’t know whether my wife have or have no HIV in her blood |
|  |  |  | - Some women do not go to facility due to fear | - all immunization helps to protect the fetus or child not to get infection from mother as well as father. |
|  |  |  | - No I don't know about the services | - Yes there is a program known as PMTCT Option B+ service and we knew it through our women who are attending this program after backing from the facility. And they show to us what they provided with there from health facility. |
|  |  |  | - there is no women who use PMTCT Option B+ here in our community | - In my opinion I and men in this community do not know further more about those services but it is given for take care of health of the mother, father, and the fetus |
|  |  |  | - I don’t know much if I have to visit health facility | - nobody knows unless those providing the services. |
|  |  |  | - No they don't know about the services | - nless those of providers who are serving the people, we don’t know such type of the service. Even we don’t know those who are using the program because they are not telling to us. |
|  |  |  | - I do not think people in this community know about HCT and PMTCT and ART. I do not know but heard | - Because I don't have much knowledge about ANC I didn't also know what is expected to do from me |
|  |  |  | - We do not know about HCT, PMTCT and Option B+. No one has told us. Not only women but husbands do not know about these | - I myself previously I didn't know whether there are such kind of services but recently I knew them through my wife. |
|  |  |  | - I do not know and my husband does not know too but we hear there are women who are using the service | - Me I knew from health care providers that there are services providing to women who are pregnant |
|  |  |  |  | - I knew that they are the services given to mothers and to their fetus to keep their status healthy. |
|  |  |  |  | - Such kinds of services are giving by this facility. Five out of ten use this services after they have identified that their test result show HIV+. |
| Manifest – Health professionals argued that people do not know about PMTCT. If men know about PMTCT, this was argued to be through their wife. Women as well as men participants commonly argued of lack of information about PMTCT. Those who heard about it heard this from health professionals at health facility level. There is lack of awareness on PMTCT although visit to health facility during pregnancy helps to prevent HIV infection. There are as few as ne out of ten women who are concerned about visiting health facility as this may affect the health of child. Although majority felt health of the mother, father, and the fetus will be protected.  Latent – There is limited awareness at all levels about PMTCT. The fact that women are not visiting health facility if ‘healthy’ and source of information is those in the facility. As a result, there is limitation of information. | | | | |
| Barriers to role of men | - most of the time what they can do are provision of food and cloths | - During delivery men buy clothes for the new born | - If my husband is not making me happy or love me, even if I or my fetus got sick, I don’t like to go to health facility | - usually gives or show love to my wife and also expend money to her for buying different things |
|  | - Men are not active in supporting their women during pregnancy due to lack of awareness | - Men are not active in supporting their women during pregnancy mainly since they are busy with other activties | - Men in our community have no more awareness what to do for their wife | - At individual level men are not active to support their wife due to alcoholism meaning he may forget what to do for his wife |
|  | - Men as husband usually they prepared themselves to buy items during delivery | - In the community men are expected to buy cloth, ensure availability of food for women after delivery | - According to existing tradition nothing can prevent the women to attend programs in health care facility | - even if men they loved their wives, if there is nothing in their hands, they cannot afford anything to buy for ladies |
|  |  |  | - support their wife to care for food and health by expending money even for their children | - in nature work such as collection of woods, food processing, making cleanliness of indoor and outdoor, feeding children…are mostly taken as women duties |
|  |  |  | - Men are not supporting their wives if the pregnancy is unwanted pregnancy | - harvesting, house and fence building, hunting….are taken as men work |
|  |  |  | - And also they are not supporting their wives due to poverty. | - It would be shame for men to support their wife by doing the above mentioned activities even if they need to support them during pregnancy. |
|  |  |  | - Lack of informaiton and awareness is a major barrier | - Men are inactive to support their wives due to weakness of work and also when they are not loved their wives so much in their hearts |
|  |  |  | - Men and women have different roles and support by men is to buy food, cloth and prepare the house | - Husband and health professions are responsible for the care of pregnant women |
|  |  |  | - Women do not go to facility due to lack of awareness | - Most women they don’t want to go to health facility because of fear of abortion in relation to drugs sometimes they will provide with. For example last time in Jor wereda one health care provider told to ladies who are pregnant in order to go to HF for medical follow up. During this time one lady refused not to go because she was convinced that if she will attend immunization she will abort her fetus |
|  |  |  | - Men are active in supporting their partners due to lack of awareness and also problem of alcoholism | - If she become aware she can go herself to health care facility to get the services like HCT and ANC lastly she will deliver at health facility safely. |
|  |  |  |  | - At individual level men are not active to support their wife due to alcoholism. |
|  |  |  |  | - Being poor for example lack of money and others, leaving far away from the place where the women are living. |
|  |  |  |  | - here should be creation of awareness meaning it will be good if people or community provided with the information about the role of men in supporting their women |
|  |  |  |  | - ou will tell her to follow immunization but most of the times our ladies are not usually accepted what we told to them for these reason will be accuse them if any problem occurred during labor or delivery |
|  |  |  |  | - but she will not respected what she have been told meaning she will do what she told not to do when you are not around her. |
|  |  |  |  | - Most women they don’t want to go to health facility because of rejection of the idea |
|  |  |  |  | - Feeling of shames to be immunize or serve with other services by health care providers mostly at early stage of pregnancy |
|  |  |  |  | - At individual level men are not active to support their wife if there are problems (disagreement) with their women i.e. for example if she is not obey what is ordered by their husband to do |
|  |  |  |  | - And culturally women’s works are not doing by men for example indoor and outdoor environmental cleanliness, food preparation |
|  |  |  |  | - On behalf of culture as told by other guy in the above, if your wife always insulting you, what you advise her to stop she is not hearing you at you will lose love and believing her. Due to this reason most men are not supporting their wives |
|  |  |  |  | - This is because in culture you cannot send back your wife to her family home in order her family to care for her health this is not good |
|  |  |  |  | - I am saying this because in culture all household thing is taken as belong to man who have power to rule these. |
|  |  |  |  | - t individual level men are not active to support their wives due to lack of knowledge |
|  |  |  |  | - t social level men are not support their wives due to social division of work i.e. indoor work is for women but not for men |
|  |  |  |  | - Men are not doing indoor works because if they do other people take as shame to them |
|  |  |  |  | - I am not supporting my wife due to lack of time for me. |
| Manifest – Men are responsible for economic support to the family, love and provide money when/if women wants to visit health facility. Traditionally, hunting, availing food, buying clothes are role of women. They are busy with this and they do not have any role to take wife to health facility.  Latent – Among the Nuer, men are not expected to take women to health facility even if men are called up on. Culturally, men are tasked for these although lack of awareness about the need for men to visit health facility along their pregnant women. | | | | |

| Care for women | - Husband, mother or neighbors are responsible for women and fetus health care | - They think nothing but pregnant mother needs care for her health | - Health professionals are expected to care for women and fetus health | - Yes there are some women attending PMTCT services. These women disclosed themselves but do not get support such as food, shelter, clothes, money |
| --- | --- | --- | --- | --- |
|  |  | - At household level husband is responsible for women and fetus health care | - During delivery he buy cloths, soaps.... to me | - Husband is responsible for the care of pregnant woman. |
|  |  |  | - Husband and neighbors are expected to care for women and fetus health. | - Health professionals are responsible |
|  |  |  | - It will be better if men fulfill our needs for example cloth, food and money | - Family of husbands have their own responsibility to care for pregnant women |
|  |  |  | - Husband and neighbors are expected to care for women and fetus health | - At primary level the one to take care of pregnant woman is husband. |
|  |  |  |  | - Since the time she released from her family, husband is responsible for all kind of her problems for example if she lack any thing to eat, to wear or if she become sick |
|  |  |  |  | Most women they don’t want to go to health facility because of fear or shame to see or examine by care providers |
| Manifest - Men are responsible for feeding and buying cloth to wife and new born. Neighbors and the community at large feel responsible to care for pregnant women  Latent – Since pregnancy is adored and is source of happiness not only for the husband and wife but also the community at large and kin groups, every community members feel responsible to support and care for women. However, it was not clear on what type of care and support community members provide. Based on the mandate of men however, providing as much love, availing food and buying clothe are identified as manifestation of care by husband. | | | | |
